# Advances in the study of traditional Chinese medicine affecting bone metabolism through modulation of oxidative stress

**DOI:** 10.3389/fphar.2023.1235854

**Published:** 2023-11-01

**Authors:** Jiaying Li, Hong Cao, Xuchang Zhou, Jianmin Guo, Chengqiang Zheng

**Affiliations:** ^1^ School of Sports and Health, Chengdu University of Traditional Chinese Medicine, Chengdu, China; ^2^ School of Kinesiology, Shanghai University of Sport, Shanghai, China; ^3^ School of Sport Medicine and Rehabilitation, Beijing Sport University, Beijing, China

**Keywords:** oxidative stress, osteoblast, osteoclast, bone metabolism, traditional Chinese medicine

## Abstract

Bone metabolic homeostasis is dependent on coupled bone formation dominated by osteoblasts and bone resorption dominated by osteoclasts, which is a process of dynamic balance between bone formation and bone resorption. Notably, the formation of bone relies on the development of bone vasculature. Previous studies have shown that oxidative stress caused by disturbances in the antioxidant system of the whole organism is an important factor affecting bone metabolism. The increase in intracellular reactive oxygen species can lead to disturbances in bone metabolism, which can initiate multiple bone diseases, such as osteoporosis and osteoarthritis. Traditional Chinese medicine is considered to be an effective antioxidant. Cumulative evidence shows that the traditional Chinese medicine can alleviate oxidative stress-mediated bone metabolic disorders by modulating multiple signaling pathways, such as Nrf2/HO-1 signaling, PI3K/Akt signaling, Wnt/β-catenin signaling, NF-κB signaling, and MAPK signaling. In this paper, the potential mechanisms of traditional Chinese medicine to regulate bone me-tabolism through oxidative stress is summarized to provide direction and theoretical basis for future research related to the treatment of bone diseases with traditional Chinese medicine.

## 1 Introduction

Bone is a hard connective tissue composed of cells, collagen fiber, and mineralized matrix. Normal bone metabolism relies on the coupled functions of bone formation and bone resorption. Bone formation is primarily dominated by osteoblasts, while bone resorption is dominated by osteoclasts. Therefore, the functional state between osteoblasts and osteoclasts determines the stable structure and function of the skeleton, known as bone homeostasis (Ramesh et al., 2021). A prerequisite for bone formation is neovascularization. The bone vascular system not only provides the necessary growth factors, hormones, and cytokines for bone formation, but also removes metabolic wastes, and also serves as a bridge between the bone and the surrounding tissues. Thus, the process of bone formation is closely related to vascularization, and bone angiogenesis also plays an important role in the regulation of bone metabolism. Oxidative stress is a complex biological process characterized by the excessive production of reactive oxygen species (ROS) in cells or tissues, leading to an imbalance in oxidative-reductive status (Ebrahimi et al., 2022). ROS are byproducts of normal metabolic processes in the body and can be classified into free radicals and non-radicals. As important signaling molecules in the body, ROS primarily function in signal transduction, molecular regulation, and other vital physiological processes (Santos et al., 2016). Under normal physiological conditions, ROS are involved in normal cellular activities at relatively low concentrations. However, under stress conditions, the body generates excessive ROS, triggering oxidative stress reactions, which can lead to cell damage or death, increased inflammation, and oxidative damage to various organs (Zou et al., 2017). Almost all metabolic processes in the body, such as glucose metabolism, lipid metabolism, and bone metabolism, are influenced by oxidative stress. The main targets of oxidative stress are proteins and nucleic acids, which affect various aspects of organismal function through multiple levels and mechanisms, including cell apoptosis and necrosis (Zhao et al., 2021). Mitochondria, as the primary cellular organelles generating ROS, are directly damaged by the excessive ROS at the subcellular level. This stimulation further triggers the release and production of ROS from mitochondria, forming a vicious cycle. Ultimately, it results in mitochondrial dysfunction, which disrupts bone formation by osteoblasts (Li et al., 2021). Current research has found a close correlation between oxidative stress caused by disturbances in the antioxidant system of the whole organism and abnormal bone loss (An et al., 2016; Schroder, 2019a), as well as the occurrence and development of various bone metabolism disorders, such as osteoarthritis (OA) and osteoporosis (OP) (Liu et al., 2022). Excessive production of ROS can significantly reduce the number and activity of osteoblasts, thereby weakening their functionality and leading to the occurrence and progression of osteoporosis (Qin et al., 2019). Furthermore, in the cartilage of patients with joint disorders, the levels of inflammatory mediators are highly elevated, leading to excessive production of ROS. Elevated ROS levels further contribute to increased mitochondrial apoptosis in chondrocytes, thereby exacerbating the progression of OA (Ansari et al., 2020; Qin et al., 2020). Additionally, ROS can stimulate the production of interleukins (such as IL-1, IL-6, and IL-7) within the body, further enhancing the expression of receptor activator of nuclear factor kappa-B (RANK) on osteoclast precursors, promoting osteoclast proliferation, and disrupting bone homeostasis (Kong et al., 2017). Moreover, oxidative stress can impair endothelial function, thereby inhibiting bone vascularization (Brito et al., 2015). Bone formation and bone vascular endothelial cell production are coupled in bone metabolism and they promote each other. Vascular endothelial cells can secrete a number of osteogenic factors to promote bone formation, while osteoblasts and other cells can also express pro-angiogenic factors to promote bone angiogenesis. Increased bone angiogenesis provides more nutrients to localized bone tissue and carries away more unfavorable metabolites.

Bone metabolic diseases, especially OP, have a serious impact on people’s lives. OP leads to an increased risk of fragility fractures in the elderly, resulting in high mortality rates and a heavy economic burden. According to incomplete statistics, nearly 100,000 people in the European Union suffer from OP, resulting in nearly 30,000 fractures per year (Barnsley et al., 2021; Yao et al., 2023). The global economic and health burden of the disease is increasing with the rapid increase in the aging population worldwide (Quicke et al., 2022; Azeez, 2023). In addition, OA causes disability and affects 50 billion people worldwide.

The current common clinical treatment for bone metabolic disorders is the use of bisphosphonates in combination with osteonutrients. However, bisphosphonates still have limitations and side effects such as osteonecrosis of the jaw and atypical femur fractures. Studies have shown that OP in postmenopausal women can be treated with estrogen, but estrogen can produce life-threatening complications such as vein thrombosis and tumors. Therefore, the search for antioxidants to inhibit oxidative stress-mediated bone metabolic disorders has become a trend (Qin et al., 2020). Currently, clinical drugs commonly used to treat bone metabolic diseases, such as nonsteroidal anti-inflammatory drugs (NSAIDs), glucocorticoids, estrogens, bisphosphonates, and parathyroid hormone, can cause serious adverse reactions. These adverse effects include kidney damage, gastrointestinal disturbances, sepsis, alopecia, and increased risk of endometrial and ovarian cancer (Strom et al., 2006; Danforth et al., 2007; Fan et al., 2017; Liu et al., 2018; Tu et al., 2019). In recent years, numerous studies have shown that traditional Chinese medicine possesses significant anti-inflammatory and antioxidant properties. It has gained widespread attention due to its fewer side effects compared to conventional clinical drugs. Research has demonstrated that traditional Chinese medicine can regulate oxidative stress caused by disorders in the body’s oxidative system, promote bone formation, inhibit bone resorption, modulate bone immunity, and promote bone vascularization, thereby treating bone-related diseases. Traditional Chinese medicine has thousands of years of rich experience, and compared with some chemically synthesized drugs, long-term use of traditional Chinese medicine causes fewer adverse reactions (Li et al., 2023). Most traditional Chinese medicines are natural plants with low cost and wide therapeutic benefits. Traditional Chinese medicine usually exerts its therapeutic effects through a “multi-component, multi-target, multi-pathway” model. The use of multiple traditional Chinese medicine together can reduce the adverse effects that occur when they are used alone and increase their efficacy. Common medicines with antioxidant properties can be categorized according to their chemical structure. Specifically, flavonoids such as licorice extract, peanut shell extract, and galangal extract can achieve antioxidant effects by forming chelates with metal ions or scavenging free radicals. Resveratrol, tea polyphenols and other polyphenolic natural antioxidants promote DNA damage repair. In addition, natural pigments such as beta-carotene, vitamins and their derivatives, antioxidant peptides, active polysaccharides and other extracts also have antioxidant properties. Therefore, this review summarizes the specific mechanisms of TCM in regulating oxidative stress and influencing bone metabolism, and discusses the current challenges. Specifically, it shows that traditional Chinese medicine affects bone formation by regulating oxidative stress through Nrf2/HO-1, PI3K/Akt, Wnt/β-catenin signaling pathways, and traditional Chinese medicine affects bone resorption by regulating oxidative stress through NF-κB, MAPK signaling pathways, and bone angiogenesis by regulating oxidative stress through Akt/mTOR/4EBP1, MAPK/ERK1/2 signaling pathways. It reveals the special role of traditional Chinese medicine in the treatment of bone metabolic diseases and provides new insights into the clinical treatment of bone-related diseases.

## 2 Traditional Chinese medicine influences bone formation through oxidative stress

Under physiological conditions, the body generates ROS through mitochondrial oxidative phosphorylation, and ROS are involved in regulating normal growth and development of the body. Several studies have shown that ROS can inhibit osteoblast proliferation, differentiation, and induce apoptosis. After oxidative stress produced by body aging, excessive ROS not only made BMSCs less capable of differentiating to osteoblasts, but also weakened their proliferative ability and prolonged their resting period, leading to decreased osteoblastogenesis. In a healthy state, there is a dynamic equilibrium between the body’s antioxidant system and oxidant system, and the production of ROS does not lead to oxidative stress. However, when the balance between the body’s antioxidant and oxidant systems is disrupted, excessive ROS production is induced, resulting in cellular oxidative damage, which is known as oxidative stress (Herbst, 2004; Talasila et al., 2013). ROS can directly or indirectly oxidize DNA, proteins, and lipids, thereby inhibiting the proliferation and differentiation of osteoblasts, inducing cellular damage and apoptosis, resulting in reduced bone formation and mineralization, leading to bone loss (Tao et al., 2020). ROS is considered an important risk factor that affects the activity and proliferation of osteoblasts. In response to this, research is actively underway to use antioxidants to protect osteoblasts from ROS-mediated oxidative stress damage. Numerous studies have revealed that metabolites from traditional Chinese medicine possess strong antioxidant properties, with fewer side effects and good therapeutic effects, making them widely applicable (Guo et al., 2014; Liu et al., 2017; Xi et al., 2018). This suggests that traditional Chinese medicine can participate in the regulation of bone formation through its antioxidant effects, and its specific mechanisms may be related to signaling pathways such as Nrf2/HO-1, PI3K-Akt, and Wnt/β-catenin (Table 1).

**TABLE 1:** Traditional Chinese medicine influences bone formation through oxidative stress.

Authors	Phytochemicals	Study subjects	Functions	Pathway
Zhang et al. (2012)	FO	Mouse osteogenic MC3T3-E1 cells	Promoting Nrf2 activation and regulate HO-1 expression	Nrf2/HO-1 signaling pathway
Cheng et al. (2019)	TSG	Osteoblastic MC3T3-E1 cells	Protecting MC3T3-E1 cells	Nrf2/HO-1 signaling pathway
Kim et al. (2021)	FST	Hydrogen peroxide (H_2_O_2_)-stimulated osteoblastic MC3T3-E1 cells	Protecting MC3T3-E1 cells	Nrf2/HO-1 signaling pathway
Han et al. (2018)	CGA	Dex-treated MC3T3-E1 cells	Inhibiting the downregulation of p21	Nrf2/HO-1 signaling pathway
Liu et al. (2021a)	Moringa metabolite	Rat bone marrow mesenchymal stem cells after peroxidative injury	Regulating FoxO1 and pAkt	PI3K/Akt signaling pathway
Zhao et al. (2022)	leonurine	Bone marrow-derived mesenchymal stem cells (BMSCs) under oxidative stress	Activating mitochondrial autophagy	PI3K/Akt signaling pathway
Li et al. (2019)	Isobavachalcone	Hydrogen peroxide (H_2_O_2_)-stimulated osteoblastic OB-6 cells	Upregulates the protein expression of tankyrase and β-catenin	Wnt/β-catenin signaling pathway
Ma et al. (2018)	OP-D	Rabbit osteoblasts incubated on titanium	—	Wnt/β-catenin signaling pathway
Gan et al. (2023)	Naringin	Mouse osteogenic MC3T3-E1 cells	Increasing Runx2 and Osterix expression	Wnt/β-catenin signaling pathway

### 2.1 Nrf2/HO-1 signaling pathway

Increasing evidence suggests that under conditions such as aging, disease, or medication, oxidative stress caused by ROS inhibits the activation of the Nrf2/HO-1 signaling pathway, thereby promoting the occurrence and progression of osteoporosis (Chen et al., 2014; Schmid et al., 2015; Weaver et al., 2016). Nuclear factor erythroid2-related factor 2 (Nrf2), as a transcription factor, has been reported to regulate the expression of antioxidant-related genes by binding to antioxidant response elements (Torrente and DeNicola, 2022). Nrf2 can bind to the antioxidant response element (ARE) in the cell, thereby regulating the cell’s antioxidant response. Under normal conditions, Nrf2 is located in the cytoplasm. In oxidative stress conditions, Nrf2 translocates into the nucleus, initiating the transcription of downstream target genes to exert its role in antioxidant stress and prevent cell apoptosis. Nrf2 plays an important role in the regulation of bone homeostasis in osteoblasts, osteoclasts and other bone cells. The role of Nrf2 in bone is complex and is influenced by a variety of factors, such as its expression level, age, gender, various physiological and pathological conditions, and its interactions with certain transcription factors that maintain normal physiological function of bone tissue. Activation of Nrf2 directly promotes osteoblast differentiation by inhibiting oxidative stress, resulting in increased bone mass. Numerous studies have shown that traditional Chinese medicine can activate Nrf2 to increase the activity of antioxidant enzymes, playing an important role in reducing oxidative damage and promoting osteoblast generation (McMahon et al., 2014; Vurusaner et al., 2016).

Crassostrea gigas. [Ostreidae] is a traditional Chinese medicine (Ihn et al., 2019). Recent studies have shown that fermented metabolite of *C. gigas. [Ostreidae]* (FO) have antioxidant effects (Molagoda et al., 2019). FO can inhibit H_2_O_2_-induced caspase-dependent apoptosis in MC3T3-E1 osteoblasts mediated by mitochondria by activating Nrf2 (Zhang et al., 2012). It was observed that suppressing Nrf2 expression through siRNA resulted in the inhibition of heme oxygenase-1 (HO-1) expression and significantly eliminated the anti-apoptotic effects of FO. It is therefore hypothesized that FO exerts its anti-oxidative stress effects and protects MC3T3-E1 cells from oxidative stress-induced apoptosis by promoting Nrf2 activation and regulating HO-1 expression. This conclusion has also been confirmed in the traditional Chinese medicine, Reynoutria multiflora (Thunb.) Moldenke [Polygonaceae] (Zhang et al., 2012). One of the important metabolites of R. multiflora (Thunb.) Moldenke [Polygonaceae] is 2,3,5,4′-tetrahydroxy-stilbene-2-O-β-D-glucoside (TSG). Research has found that TSG exerts antioxidant effects through the Nrf2/HO-1 signaling pathway to alleviate H_2_O_2_-induced oxidative damage in osteoblasts. Cheng et al. (Cheng et al., 2019) investigated the effects of H_2_O_2_ on the expression of Nrf2 and its downstream genes HO-1 and NAD(P)H: quinone oxidoreductase 1 (NQO1). The results showed that H_2_O_2_ treatment significantly decreased the expression levels of Nrf2, HO-1, and NQO1, while intervention with TSG could significantly reverse these changes. After H_2_O_2_ treatment, compared to the negative control, the expression of apoptotic markers caspase-3, caspase-9, and Bax significantly increased, while the expression of the anti-apoptotic marker Bcl-2 significantly decreased. However, treatment with TSG could also significantly reverse these changes. In summary, TSG can protect MC3T3-E1 cells from oxidative stress-induced caspase-mitochondrial apoptosis. The mechanism of action may involve the activation of the Nrf2/HO-1 signaling pathway, which exerts antioxidant effects (Cheng et al., 2019). Data shows that in the presence of H_2_O_2_, the important metabolites Fermented Sea T angle (FST) of the traditional Chinese medicine Saccharina japonica [Phaeophyceae] significantly promotes nuclear translocation and phosphorylation of Nrf2, accompanied by upregulation of HO-1 expression. Kim et al. (Kim et al., 2021) further confirmed that FST inhibits oxidative stress through the Nrf2/HO-1 pathway by pre-treating MC3T3-E1 cells with the HO-1 inhibitor zinc protoporphyrin (ZnPP) to block the Nrf2/HO-1 signaling pathway. Studies have shown that pre-treatment with ZnPP significantly abolishes the protective effect of FST on H_2_O_2_-induced damage in MC3T3-E1 cells. Therefore, these results suggest that FST can protect MC3T3-E1 cells from H_2_O_2_-induced damage and apoptosis by preserving mitochondrial function, scavenging ROS, and activating the Nrf2/HO-1 antioxidant pathway. Additionally, research has also found that chlorogenic acid (CGA) can promote osteoblastogenesis through the activation of the Nrf2/HO-1 pathway. The specific mechanism may involve the regulation of p21 (Han et al., 2019). CGA is a polyphenolic metabolite formed by the esterification of caffeic acid and quinic acid. It is a major active metabolite in many traditional Chinese medicines, such as Lonicera japonica Thunb. [Caprifoliaceae] and Eucommia ulmoides [Eucommiaceae] (Han et al., 2018). P21 (also known as p21Waf1/Cip1) is a multifunctional cell cycle regulatory molecule that mediates the interactions of various proteins in multiple signaling pathways. CGA promotes the Nrf2/HO-1 pathway by inhibiting the downregulation of p21, thereby reducing ROS generation and enhancing the antioxidant capacity of MC3T3-E1 cells (Toledano, 2009). The results demonstrate that CGA treatment significantly increases nuclear Nrf2 and p21 levels and upregulates HO-1 protein expression, indicating that the antioxidant function of p21 is mediated through the activation of Nrf2 (Toledano, 2009).

In summary, traditional Chinese medicines such as C. gigas. [Ostreidae], S. japonica [Phaeophyceae], and L. japonica Thunb. [Caprifoliaceae] have been shown to upregulate Nrf2 levels, thereby activating the Nrf2/HO-1 pathway to inhibit ROS generation in MC3T3-E1 cells and protect osteoblasts from oxidative stress-induced cell apoptosis. Under oxidative stress conditions, ROS can induce phosphorylation of Nrf2, leading to dissociation of Nrf2 from Kelch-like ECH-associated protein 1 (Keap1), resulting in reduced ubiquitination of Nrf2. This allows more activated Nrf2 to translocate into the nucleus, form heterodimers with Maf proteins, and bind to ARE to initiate the transcription of HO-1 gene (McCubrey et al., 2006). However, current research on traditional Chinese medicines mainly focuses on the regulation of downstream molecule HO-1 after Nrf2 nuclear translocation, and further studies are needed to explore the role of the upstream signaling pathways involved in Nrf2 phosphorylation.

### 2.2 PI3K/Akt signaling pathway

The PI3K/Akt signaling pathway is a classical pathway involved in anti-apoptosis and cell survival. It has been shown to participate in the regulation of intracellular ROS levels and cellular fate, playing a crucial role in cell survival (Le Belle et al., 2011). The PI3K/Akt signaling pathway is considered to be important in the osteogenic differentiation of bone marrow mesenchymal stem cells (BMSCs) (Carracedo et al., 2008). Meanwhile, the PI3K/Akt signaling pathway is an important regulator of chondrocyte survival and apoptosis. Among them, Akt and its downstream target protein, mammalian target of rapamycin (mTOR), are critical regulatory factors in bone formation, is also a key inhibitor of autophagy, centrally regulated by an upstream signaling pathway involving PI3K/Akt (Sun et al., 2020), playing a key role in the process of osteogenesis (Yang et al., 2013). When activated, Akt and its target genes promote osteoblast differentiation and mineralization. Meanwhile, when PTEN, an endogenous inhibitor of PI3K, is deficient, the Akt signaling pathway is significantly enhanced and bone mass is increased.

Moringa oleifera Lam. [Moringaceae] is an angiosperm, and different parts of the plant have been used throughout history as food and traditional medicine (Stohs and Hartman, 2015). Liu et al. (Liu et al., 2021a) evaluated the effects of M. oleifera metabolite on the expression levels of transcription factor FoxO1 (Fork head Box O1) and pAkt (two downstream targets of the PI3K/Akt pathway). The experiments initially observed that Moringa metabolite could promote osteogenic differentiation and reverse the decrease in Foxo1 caused by oxidative damage, as well as prevent apoptosis in BMSCs. Further investigation revealed that M. oleifera leaf metabolite exerted its effects by increasing the p-Akt/Akt ratio, promoting Akt phosphorylation, activating the PI3K/Akt/Foxo1 pathway, and thereby reducing H_2_O_2_-induced oxidative damage, inhibiting BMSC apoptosis, and promoting osteogenic differentiation. Additionally, in murine pre-osteoblast MC3T3-E1 cells, the PI3K/Akt signaling pathway has also been demonstrated to promote osteogenic differentiation (Fujita et al., 2004).

Mitochondria are the main source of ROS generation. Impairment of mitochondrial function can lead to excessive ROS production, resulting in oxidative stress. Restoring mitochondrial function to maintain the antioxidant and oxidative balance of the organism can effectively exert antioxidant stress and resist oxidative stress-induced cell apoptosis. Mitophagy, the targeted engulfment and degradation of mitochondria by the cellular autophagic machinery, is generally regarded as the primary mechanism for mitochondrial quality control, in which the PI3K/Akt signaling pathway is a classical pathway mediating mitophagy (Palikaras et al., 2018). Zhao et al. (Zhao et al., 2022) found that restoration of mitochondrial function with traditional Chinese medicine can attenuate ROS-induced damage to BMSCs (Zhu et al., 2018). Leonurine, a natural metabolite from the traditional Chinese medicine.

Leonurus japonicus Maxim.[Lamiaceae], was investigated. The study showed that the appropriate concentration of leonurine can alleviate oxidative stress-induced apoptosis in rat BMSCs and promote their osteogenic differentiation capacity. *In vitro* studies also demonstrated that leonurine can improve bone healing in ovariectomized (OVX) rats. Additionally, treatment with a PI3K activator (740-YP) partially inhibited leonurine-induced mitophagy. The biological informatics analysis suggests that leonurine strongly binds to the PI3K protein at the Asp841, Glu880, and Val882 sites. It can be inferred that leonurine inhibits the PI3K/Akt pathway by directly binding to PI3K, thereby activating mitochondrial autophagy in BMSCs to protect them from excessive ROS-induced damage and apoptosis.

In summary, traditional Chinese medicines such as M. oleifera Lam. [Moringaceae] and L. japonicus Maxim.[Lamiaceae] regulate oxidative stress through the PI3K/Akt signaling pathway. The traditional Chinese medicine can reduce ROS levels, alleviate oxidative stress-induced apoptosis in BMSCs, and promote osteogenic differentiation of BMSCs. However, the current knowledge of traditional Chinese medicine in this context is limited, and further research is needed to explore whether other traditional Chinese medicine can also regulate oxidative stress through the PI3K/Akt signaling pathway.

### 2.3 Wnt/β-catenin signaling pathway

The Wnt/β-catenin signaling pathway is a key pathway involved in osteoblast differentiation, regulating the formation of osteoblasts and playing a crucial role in bone regeneration and remodeling (Burgers and Williams, 2013; Duan and Bonewald, 2016). The canonical Wnt signaling pathway exerts its biological activity by upregulating the activity of intracellular β-catenin. In the absence of Wnt signaling, a complex consisting of glycogen synthase kinase-3β (GSK3β), Axin, and adenomatous polyposis coli (APC) phosphorylates and inactivates β-catenin, thereby inhibiting osteogenesis (Martinez-Gil et al., 2022). When Wnt proteins bind to receptors on the cell membrane, cytoplasmic scaffold protein disheveled (Dvl) is phosphorylated. Phosphorylated Dvl, in turn, inhibits the binding of the GSK3β complex to β-catenin, allowing free β-catenin to accumulate in the cytoplasm and enter the nucleus, where it binds to transcription factors TCF/LEF, activating Wnt target genes such as COX-2 and c-Myc, thereby promoting osteoblast proliferation and differentiation (Clevers and Nusse, 2012). Currently, it has been discovered that the Wnt/β-catenin signaling pathway also plays a key role in the process of oxidative stress affecting bone metabolism. Oxidative stress is a major cause of age-related bone loss and can lead to a decrease in the number of osteoblasts and bone formation. β-catenin has been widely used in recent years as a major factor in the regulation of oxidative stress. Numerous studies have shown that traditional Chinese medicine can activate the Wnt/β-catenin pathway to alleviate oxidative stress-induced damage to osteoblasts, thereby upregulating bone formation.

Isobavachalcone is a natural tricyclic aromatic metabolite. *In vitro* experiments were conducted on OB-6 osteoblast cells using H_2_O_2_ or H_2_O_2_^+^isobavachalcone treatment to assess cell viability, apoptosis, ROS production, and calcium accumulation. The results showed that H_2_O_2_ treatment reduced cell viability, expression levels of Runt-related transcription factor 2 (Runx2) and osteocalcin (OCN), as well as calcium deposition. Furthermore, it significantly increased cell apoptosis and ROS production (Li et al., 2019). However, isobavachalcone exhibits significant protective effects against the decreased functionality and viability of osteoblasts induced by H_2_O_2_. Moreover, isobavachalcone effectively upregulates the protein expression of tankyrase and β-catenin, the key transduction factors of the Wnt/β-catenin pathway, thereby inhibiting excessive ROS generation and protecting OB-6 cells from H_2_O_2_-induced mitochondrial damage (Li et al., 2019). In summary, current research results demonstrate that isobavachalcone attenuates oxidative stress-induced damage in osteoblasts through the Wnt/β-catenin signaling pathway, suggesting it may be a novel therapeutic approach for osteoporosis. Building upon these findings, Ma et al. (Ma et al., 2018) implanted titanium alloy implants into the femoral condyles of diabetic rabbits and treated them with Ophiopogonin D (OP-D), a metabolite found in dwarf lilyturf tuber. They demonstrated that OP-D can activate the Wnt/β-catenin signaling pathway to alleviate oxidative stress response, improve osteoblast proliferation and differentiation in diabetic rabbits, mitigate apoptosis damage, and thus reverse the impaired functionality of osteoblasts (Jaschke et al., 2020). The study further demonstrated that OP-D exerts its role in clearing ROS and improving the biological function of osteoblasts by activating the Wnt/β-catenin pathway, as evidenced by the inhibition of the Dkk1 molecule, which is involved in suppressing the Wnt/β-catenin pathway.Similarly, naringin (4′,5,7-trihydroxy flavanone-7-rhamnoglucoside) ameliorated H_2_O_2_-induced inhibition of osteogenic differentiation by activating the Wnt/β-catenin signaling pathway in human adipose stromal cells (ADSC). Naringin is an extract of the Chinese medicine Citrus maxima (Burm.) Merr. [Rutaceae], which is widely used in traditional Chinese medicine for the treatment of OP. Studies have shown that naringin rescues H_2_O_2_-inhibited β-catenin and cyclin D1 expression by inhibiting oxidative stress, stimulates alkaline phosphatase (ALP) activity, increases Runx2 and Osterix expression, and promotes osteogenic differentiation by inhibiting oxidative stress (Gan et al., 2023).

In summary, traditional Chinese medicines such as Cullen corylifolium (L.) Medik.[Fabaceae], Ophiopogon japonicus (Thunb.) Ker Gawl. [Asparagaceae] and C. maxima (Burm.) Merr. [Rutaceae], exert their protective effects on osteoblasts against oxidative stress-induced mitochondrial apoptosis by acting on the key transduction factors of the Wnt/β-catenin signaling pathway, thus reducing ROS generation. Considering that the Wnt/β-catenin signaling pathway involves multiple transcription factors such as TCF/LEF, enzymes like casein kinase 1 (CK1), and GSK3β, further research is needed to investigate whether these proteins and enzymes can be influenced by other traditional Chinese medicine. Additionally, since the Wnt signaling pathway regulates the multipotent differentiation of stem cells and bone development and regeneration, it is also necessary to explore the crosstalk between this pathway and other signaling pathways that can impact bone formation. For example, *C. corylifolium* in kidney tonic botanical drugs can not only increase the protein levels of Smad1, Smad5 and Smad8 and the expression of Osterix, which promotes osteogenic differentiation of osteoblasts through BMP-Smads, but also upregulate the expression of β-catenin, which is involved in the activation of Wnt/β-catenin signaling pathway (Ren et al., 2020). Therefore, the interconnections between signaling pathways of TCM affecting bone formation through oxidative stress should be one of the hotspots and focuses of future research.

## 3 Traditional Chinese medicine affects bone resorption through oxidative stress

**FIGURE 1:**
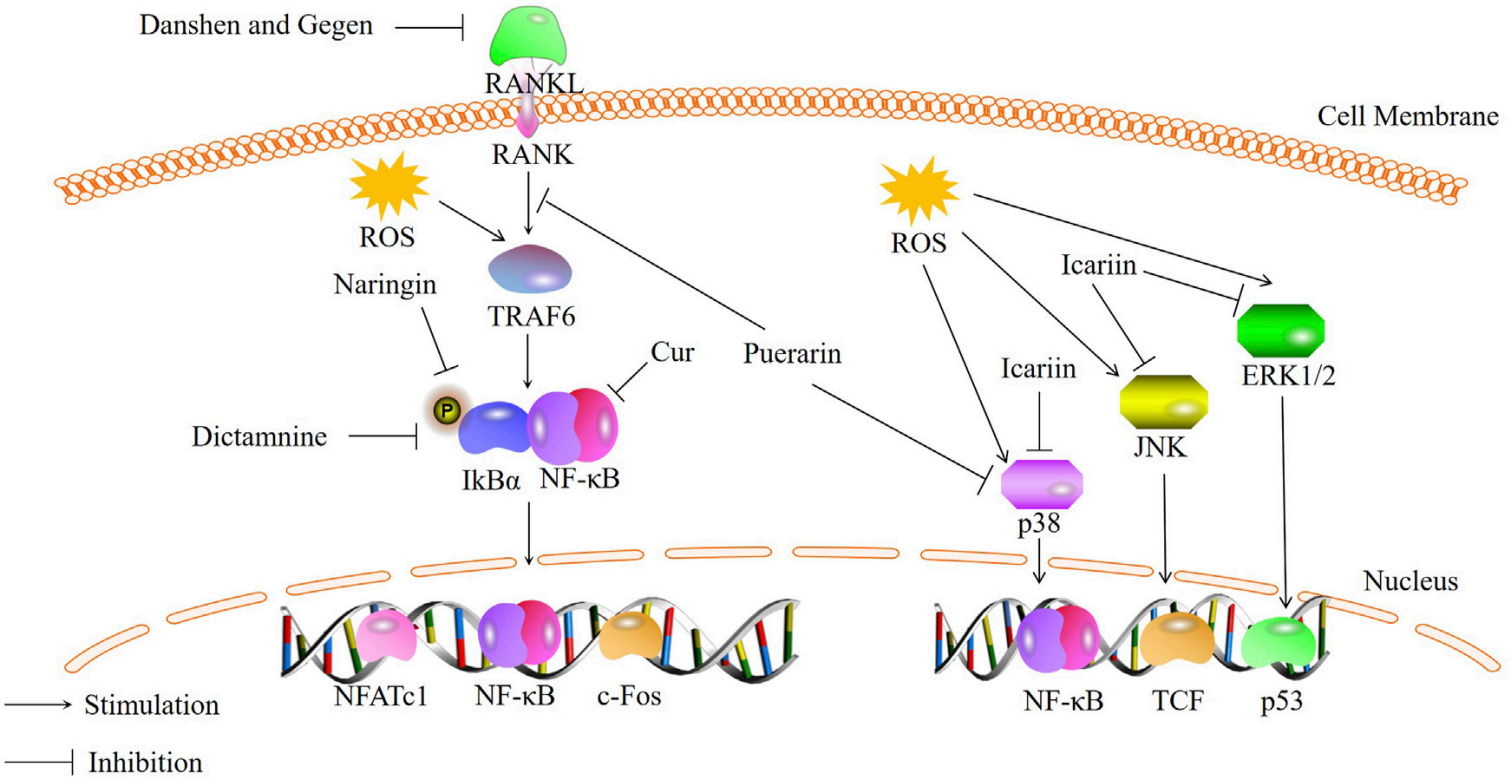
Bioactive metabolites inhibit osteoclastogenesis through NF-κB and MAPK signaling pathways.

Osteoclasts (OCs) are multinucleated cells primarily responsible for bone resorption (Ono and Nakashima, 2018). Normal bone metabolism is mediated by the coordinated activities of bone resorption and bone formation. Excessive bone resorption can lead to disrupted bone metabolism and a variety of bone diseases (Liu et al., 2021b). The formation and function of OCs are regulated by various cytokines, including macrophage colony-stimulating factor (M-CSF) and receptor activator of NF-κB ligand (RANKL) secreted by osteoblasts. RANK, located on the surface of osteoclast precursor cells, can bind to RANKL secreted by osteoblasts. This binding activates a series of transcription factors, including nuclear factor κB (NF-κB) and mitogen-activated protein kinase (MAPK)-related cytokines, resulting in increased expression and translocation of nuclear factor of activated T-cells, cytoplasmic 1 (NFATc1) from the cytoplasm to the nucleus. The expression of the aforementioned osteoclast-related factors can induce the differentiation of osteoclast precursors into mature OCs and promote the expression of genes associated with osteoclast formation and bone resorption, including tartrate-resistant acid phosphatase (TRAP), cathepsin K (CTSK), and matrix metalloproteinase-9 (MMP-9) (Huang et al., 2017; Wong et al., 2022). Increasing evidence suggests a relationship between oxidative stress and osteoclast formation (Kim et al., 2020). With aging and estrogen deficiency, ROS accumulate in the skeleton (Manolagas, 2010), and excessive ROS production can activate various signaling pathways and increase bone resorption in the body, accelerating bone loss (Kim et al., 2017). Therefore, inhibiting oxidative stress-induced osteoclast formation has become a key focus in the treatment of bone metabolic disorders. Recently, traditional Chinese medicine has gained increasing attention due to its low toxicity and high safety in long-term treatment (Adler et al., 2016; Kelly et al., 2019). Numerous studies have shown that traditional Chinese medicine can regulate oxidative stress through signaling pathways such as NF-κB and MAPK, thereby inhibiting excessive bone resorption (Figure 1).

### 3.1 NF-κB signaling pathway

NF-κB is a class of inducible transcription factors that regulate various cellular behaviors, including bone inflammation, osteoblast growth, and apoptosis. It is typically present in an inactive state as a p50/p65 heterodimer and its activity is regulated by intracellular oxidative stress levels. ROS act as mediators during RANKL-induced osteoclast differentiation and can control the translocation of the NF-κB transcription factor to the cell nucleus (Callaway and Jiang, 2015). Activation of the NF-κB signaling pathway can accelerate chondrocyte apoptosis, leading to increased bone resorption and resulting in diseases such as OA and synovitis (Choi et al., 2019). Various stimuli can activate the NF-κB signaling pathway, such as tumor necrosis factor-alpha (TNF-α), lipopolysaccharide (LPS), interleukin-1 beta (IL-1β), and it can regulate the transcription of many genes, including pro-inflammatory cytokines and chemokines such as IL-6, cell cycle genes such as cyclin D1, anti-apoptotic genes such as Bcl-2, and MMP3 (matrix metalloproteinase 3) (Yabas et al., 2021). There are two cascades of reactions that can activate NF-κB, and both involve the activation of an IκB kinase (IKK) complex. This complex consists of catalytic kinase subunits (IKKα and/or IKKβ) and a non-enzymatic regulatory scaffold protein called NEMO (NF-κB essential modulator, also known as IKKγ). NF-κB dimers are activated through IKK-mediated phosphorylation of IκB, which leads to the degradation of the phosphorylated IκB proteins by the proteasome. This allows the active NF-κB subunits to translocate into the cell nucleus and induce the expression of target genes. Activation of NF-κB results in the expression of the IκBα gene, which serves as a negative feedback loop to sequester NF-κB subunits and terminate signal transduction in the absence of sustained activating signals (Gilmore, 2006; Scheidereit, 2006). It has been reported that traditional Chinese medicine can inhibit the NF-κB signaling pathway, thereby reducing the formation of OCs induced by oxidative stress.

Curculigo orchioides (Cur), a natural phenolic glycoside metabolite, is the main metabolites of the traditional Chinese medicine Curculigo orchioides [Hypoxidaceae]. Liu et al. (Liu et al., 2021b) investigated the effects of Cur on RAW264.7 cells stimulated with RANKL and H_2_O_2_. They found that Cur can inhibit the expression and phosphorylation of p65, exerting inhibitory effects on the NF-κB signaling pathway. This leads to a reduction in excessive ROS production induced by H_2_O_2_ and osteoclast differentiation. Therefore, Cur can act as an inhibitor of osteoclast formation by inhibiting the NF-κB signaling pathway. Qin et al. (Qin et al., 2020) also demonstrated through *in vitro* studies that some of the traditional Chinese medicine can suppress osteoclast differentiation and maturation by regulating the NF-κB signaling pathway. They used a combination of Salvia miltiorrhiza Bunge [Lamiaceae] (Danshen) and *Pueraria montana* var. *lobata [Fabaceae]* (Gegen) (DG) to intervene in rats and found that the DG combination significantly reduced the expression of osteoclast-specific markers RANK, c-Fos, and NFATc1. Moreover, DG was able to preclude the activation of the NF-κB pathway induced by RANKL. Studies have shown that DG significantly inhibited RANKL-stimulated NF-κBp65 phosphorylation. DG can inhibit the activation of the NF-κB/p65 pathway, leading to a significant reduction in RANKL-induced ROS production and a notable suppression of osteoclast differentiation. Similarly, naringin is also believed to inhibit the phosphorylation of p65 and IκBα, thereby inhibiting the activation of the NF-κB signaling pathway and oxidative stress, resulting in reduced osteoclast formation in the intervertebral disc (Zhang et al., 2022). Wong et al. (Wong et al., 2022) demonstrated that traditional Chinese medicine can suppress NFATc1 expression and inhibit the NF-κB signaling pathway. Dictamnine (DIC), a novel RANKL-targeted furanquinoline alkaloid, has been shown to bind specifically to RANKL *in vitro*, inhibit the activity of Nrf2, and suppress ROS production by inhibiting NFATc1 expression and the activation of the NF-κB signaling pathway. Research has confirmed that DIC can bind specifically to RANKL and affect the NF-κB signaling pathway by inhibiting NFATc1 expression, inhibits the transcriptional activity of NFATc1 and interferes with the expression of NF-κBp65 and its phosphorylated form, thereby reducing the expression of IκBα(Clohisy et al., 2004; Suzuki et al., 2011). This inhibition of the NF-κB/IκBα pathway leads to reduced ROS production and decreased bone resorption.In summary, traditional Chinese medicine inhibits the transcription and expression of NFATc1, as well as reduces the phosphorylation of p65, thereby inhibiting the activation of the NF-κB pathway and reducing ROS production, ultimately leading to a decrease in osteoclast formation. However, there is still limited research on the downstream target proteins of the NF-κB pathway in response to traditional Chinese medicine. Further *in vivo* studies are needed to investigate this aspect.

### 3.2 MAPK signaling pathway

The MAPK signaling pathway is an essential signal transduction pathway in organisms and is involved in physiological processes such as cell growth, differentiation, and proliferation. It plays a crucial role in regulating osteoclast differentiation and inducing and activating osteoclast formation and function (Li et al., 2016; Wu et al., 2021). The MAPK signaling pathway is present in eukaryotic cells and belongs to the serine/threonine protein kinase family. Under normal physiological conditions, ROS regulate cell growth and development through the MAPK signaling pathway. However, when ROS levels become imbalanced, excessive ROS can enhance the phosphorylation of p38, ERK1/2, and JNK, contributing to processes such as inflammation and cell apoptosis (Xiao et al., 2020). In recent years, research has shown that the mechanism by which traditional Chinese medicine reduces ROS to inhibit osteoclast formation is associated with the MAPK signaling pathway.

Arctiin (ARC) is a lignan metabolite derived from the seeds of Arctium lappa L. [Asteraceae]. Recent studies have demonstrated that ARC treatment inhibits the activation of the MAPK pathway (Qin et al., 2020)and enhances the expression of antioxidant enzymes, thereby suppressing osteoclast formation *in vitro* and alleviating bone loss *in vivo*. Preclinical studies (Liu et al., 2022)have also shown that ARC has the potential to inhibit osteoclast formation by suppressing the activation of the MAPK signaling pathway induced by RANKL, thereby preventing bone loss induced by OVX. Building upon this, Xiao et al. (Xiao et al., 2020)investigated the mechanism by which puerarin attenuates osteoclast-related bone loss in an OVX-induced osteoporosis mouse model. The experiments revealed that RANKL binds to its receptor, recruiting tumor necrosis factor receptor-associated factor 6 (TRAF6) to the cytoplasmic region, subsequently activating the MAPK signaling pathway and inducing the expression of osteoclast genes. RANKL induction increases the intracellular levels of ROS and activates the MAPK signaling pathway, but puerarin significantly reduces these effects. TRAF6 is markedly increased in RANKL-induced RAW264.7 cells, but its levels decrease upon the addition of puerarin. Furthermore, Western blot analysis demonstrates that puerarin can also decrease the levels of phosphorylated ERK, JNK, and p38 in RANKL-induced RAW264.7 cells. These results suggest that puerarin inhibits osteoclast differentiation and activity, thus reducing bone loss in an ovariectomy-induced osteoporosis mouse model, through the reduction of ROS levels and ROS-dependent MAPK signaling pathway. Epimedium brevicornu Maxim.[Berberidaceae], a traditional Chinese medicine, contains a major flavonoid metabolite called icariin. Icariin can also inhibit the MAPK signaling pathway to suppress RANKL-induced osteoclastogenesis and reduce bone loss, primarily through three major signaling cascades: ERK-MAPK, JNK-MAPK, and p38-MAPK (Zhou et al., 2021). Experimental results indicate (Xu et al., 2019) that icariin can similarly inhibit the phosphorylation of these three MAPKs and the generation of ROS in RANKL-induced RAW264.7 cells. This suggests that the inhibitory effect of icariin on osteoclast differentiation may be achieved by modulating the phosphorylation of MAPKs in mature OCs and reducing ROS production (Wei et al., 2022).

In summary, traditional Chinese medicine can inhibit the phosphorylation of p38, ERK, and JNK to modulate the MAPK signaling pathway, reduce ROS, and thereby suppress osteoclast formation. Moreover, multiple studies have shown that the MAPK signaling pathway is involved in the activation of NF-κB (Huang et al., 2015; Tsumagari et al., 2015; Lan et al., 2020). Therefore, further research is needed to elucidate the specific mechanisms by which traditional Chinese medicine regulates osteoclast formation and the interplay between these two pathways.

## 4 Traditional Chinese medicine influences bone angiogenesis through oxidative stress

Research has indicated that bone formation and bone angiogenesis are closely linked processes. The correlation between bone metabolism and bone angiogenesis is not adequately described. On one hand, bone cells secrete pro-angiogenic factors, with the most important one being vascular endothelial growth factor (VEGF), which triggers signaling responses in various cell populations including endothelial cells, chondrocytes, osteoblasts, and OCs (Portal-Nunez et al., 2012). VEGF is one of the most direct target genes of hypoxia-inducible factor-1α (HIF-1α), its secretion is essential for the coupling of osteogenesis and bone angiogenesis. VEGF attracts endothelial cells to bone tissue and directly controls the differentiation and function of osteoblasts and osteoclasts involved in bone remodeling. VEGF, which is indispensable for angiogenesis in developing or mature bone tissue, is mainly derived from chondrocytes, mesenchymal stem cells, or vascular-confined endothelial cells. On the other hand, bone vascular endothelial cells release cytokines that can act on chondrocytes and osteoblast lineages, and angiogenesis also plays an important role in bone formation. Bone marrow-derived endothelial progenitor cells (BM-EPCs) are a promising alternative cellular source for promoting angiogenesis in regenerative medicine, and endothelial progenitor cells can enhance bone repair in specific locations (Rozen et al., 2009; Atesok et al., 2010). Pathophysiological evidence suggests that increased generation of ROS and oxidative stress contribute to vascular dysfunction (Zhai et al., 2014). HIF-1α is a transcription factor encoded by activated genes in tissue cells under hypoxia. Under normoxic conditions, the oxygen-dependent degradation structural domain (ODD) on its oxygen-sensing element HIF-1α is hydroxylated by the active prolyl hydroxylase (PHD), the bound VHL protein then enters the proteasome to be degraded. In contrast, in hypoxic or anoxic environments, PHD activity is lost or reduced, leading to the accumulation and translocation of HIF-1α into cells to bind to the β-subunit, which activates HIF’s target genes, such as VEGF, to act. A low oxygen environment exists to varying degrees during bone development, metabolism, fracture healing and tumors. Under hypoxic conditions, bone marrow mesenchymal stem cells (BMSCs) enhance their angiogenic properties by upregulating VEGF. Upon elevation of ROS levels, HIF-1α increases and initiates the transcription of VEGF. Therefore, disruption of ROS at normal levels would impede the transcription of VEGF, thereby affecting bone angiogenesis (Black et al., 2008; Schroder, 2019b). Based on the significant role of traditional Chinese medicine in reducing ROS, further research has discovered its notable effectiveness in the treatment of bone vascular diseases.

Salidroside (SAL), a major metabolites in Rhodiola rosea L [Crassulaceae], can enhance the proliferation and differentiation of bone marrow endothelial progenitor cells, promoting the secretion of VEGF and nitric oxide (NO), thereby facilitating the angiogenic differentiation of bone marrow endothelial cells (Tang et al., 2014). Studies have shown that SAL can also stimulate the phosphorylation of mTOR, p70S6 kinase Akt, as well as the phosphorylation of ERK1/2, which is associated with angiogenesis. Furthermore, SAL can reverse the phosphorylation of JNK and p38 MAPK induced by H_2_O_2_, inhibit the change in Bax/Bcl2 ratio after H_2_O_2_ stimulation, significantly reduce H_2_O_2_-induced cell apoptosis, lower intracellular ROS levels, and restore the mitochondrial membrane potential in BM-EPCs. The results indicate that SAL exerts antioxidant effects and promotes angiogenesis in human bone marrow endothelial progenitor cells through the Akt/mTOR/p70S6K and MAPK signaling pathways. This viewpoint is also supported by the study conducted by Tang et al. (Tang et al., 2015). We observed that treatment with icariin significantly enhanced cell migration and capillary formation in BM-EPCs. It effectively mitigated H_2_O_2_-induced cell apoptosis and autophagic programmed cell death, which was associated with reduced intracellular ROS levels and restoration of mitochondrial membrane potential. Additionally, icariin prevented endothelial cell damage caused by H_2_O_2_ by increasing NO content and decreasing caspase expression (Wang and Huang, 2005). Experimental results demonstrated that icariin can strongly promote angiogenic differentiation and cellular protection by modulating the Akt/mTOR/4EBP1, p38/ATF2, and MAPK/ERK1/2 signaling pathways, thus counteracting H_2_O_2_-induced cell apoptosis and autophagy.

In summary, traditional Chinese medicine can regulate signaling pathways such as Akt, MAPK/ERK1/2, to protect cells from oxidative stress, restore mitochondrial membrane potential in BM-EPCs, and promote bone angiogenesis. Currently, there is limited research on traditional Chinese medicine that can influence bone vascular formation, and further exploration is needed to investigate the effects of other traditional Chinese medicine on bone angiogenesis. Additionally, further research can be conducted to explore the interconnections between pathways associated with bone vascular formation.

## 5 Conclusions and perspectives

The traditional Chinese medicine can regulate oxidative stress caused by disorders of the body’s metabolic system, reduce intracellular ROS levels, and impact bone metabolism to treat or inhibit various bone diseases. Specifically, it involves activating signaling path-ways such as Nrf2/HO-1, PI3K/Akt, and Wnt/β-catenin to promote osteoblast generation and accelerate bone formation. It also involves inhibiting signaling pathways such as NF-κB, MAPK, and RANKL/RANK/OPG to reduce osteoclast formation and slow down bone resorption. Furthermore, through the Akt/mTOR/4EBP1 and MAPK/ERK1/2 signaling pathways, it enhances the proliferation and vascular differentiation of bone mar-row endothelial cells, thus promoting bone angiogenesis. However, further research is needed to explore whether traditional Chinese medicine metabolites can affect bone metabolism through other signaling pathways, such as the RANKL/RANK/OPG signaling pathway, and whether it acts on other downstream factors of the signaling pathways. Furthermore, research on the effects of traditional Chinese medicine metabolites and traditional Chinese medicine preparations on reducing ROS through regulating mitochondrial autophagy is still limited. At the same time, oxidative stress can be caused by disturbances in the body’s antioxidant system, as well as damage to bones and other tissues. But in the current study, it is almost the effect of botanical drugs on the antioxidant system of the whole body organism. Studies using OVX rats are commonly conducted, but it is not clear whether metabolites of traditional Chinese medicine can modulate other causes of oxidative stress. In future studies, it would be beneficial to focus on the effects of various traditional Chinese medicine metabolites and traditional Chinese medicine preparations on bone immunology and bone angiogenesis through oxidative stress, as well as their specific mechanisms. Additionally, exploring more traditional Chinese medicinal plants within the same botanical family that can influence bone metabolism and elucidating their specific actions would be valuable. Understanding the mechanisms of traditional Chinese medicine’s regulation of bone metabolism through oxidative stress can also be investigated by studying their effects on different target cells. Studies on whether traditional Chinese medicine preparations can modulate the destruction of bone and other tissues leading to oxidative stress also need to be further investigated. At the same time, it is necessary to carry out more in-depth research on a variety of receptors and ligands involved in the signaling pathway, growth factors, transcription factors, gene regulation, etc., to find out the specific mechanism of the action of traditional Chinese medicine metabolites and traditional Chinese medicine preparations on the signaling pathway. Furthermore, more *in vivo* experiments and clinical applications are needed to validate the effects of traditional Chinese medicine on bone metabolism disorders.

## References

[B1] Adler R. A. El-Hajj Fuleihan G. Bauer D. C. Camacho P. M. Clarke B. L. Clines G. A. (2016). Managing osteoporosis in patients on long-term bisphosphonate treatment: report of a task force of the American society for bone and mineral research. J. Bone Min. Res. 31 (10), 1910. 10.1002/jbmr.2918 27759931

[B2] An J. Yang H. Zhang Q. Liu C. Zhao J. Zhang L. (2016). Natural products for treatment of osteoporosis: the effects and mechanisms on promoting osteoblast-mediated bone formation. Life Sci. 147, 46–58. 10.1016/j.lfs.2016.01.024 26796578

[B3] Ansari M. Y. Ahmad N. Haqqi T. M. (2020). Oxidative stress and inflammation in osteoarthritis pathogenesis: role of polyphenols. Biomed. Pharmacother. 129, 110452. 10.1016/j.biopha.2020.110452 32768946 PMC8404686

[B4] Atesok K. Li R. Stewart D. J. Schemitsch E. H. (2010). Endothelial progenitor cells promote fracture healing in a segmental bone defect model. J. Orthop. Res. 28 (8), 1007–1014. 10.1002/jor.21083 20135674

[B5] Azeez T. A. (2023). Osteoporosis and cardiovascular disease: a review. Mol. Biol. Rep. 50 (2), 1753–1763. 10.1007/s11033-022-08088-4 36449152

[B6] Barnsley J. Buckland G. Chan P. E. Ong A. Ramos A. S. Baxter M. (2021). Pathophysiology and treatment of osteoporosis: challenges for clinical practice in older people. Aging Clin. Exp. Res. 33 (4), 759–773. 10.1007/s40520-021-01817-y 33742387 PMC8084810

[B7] Black S. M. DeVol J. M. Wedgwood S. (2008). Regulation of fibroblast growth factor-2 expression in pulmonary arterial smooth muscle cells involves increased reactive oxygen species generation. Am. J. Physiol. Cell Physiol. 294 (1), C345–C354. 10.1152/ajpcell.00216.2007 17942638 PMC3970933

[B8] Brito R. Castillo G. Gonzalez J. Valls N. Rodrigo R. (2015). Oxidative stress in hypertension: mechanisms and therapeutic opportunities. Exp. Clin. Endocrinol. Diabetes 123 (6), 325–335. 10.1055/s-0035-1548765 25918881

[B9] Burgers T. A. Williams B. O. (2013). Regulation of Wnt/beta-catenin signaling within and from osteocytes. Bone 54 (2), 244–249. 10.1016/j.bone.2013.02.022 23470835 PMC3652284

[B10] Callaway D. A. Jiang J. X. (2015). Reactive oxygen species and oxidative stress in osteoclastogenesis, skeletal aging and bone diseases. J. Bone Min. Metab. 33 (4), 359–370. 10.1007/s00774-015-0656-4 25804315

[B11] Carracedo A. Ma L. Teruya-Feldstein J. Rojo F. Salmena L. Alimonti A. (2008). Inhibition of mTORC1 leads to MAPK pathway activation through a PI3K-dependent feedback loop in human cancer. J. Clin. Invest. 118 (9), 3065–3074. 10.1172/JCI34739 18725988 PMC2518073

[B12] Chen K. Zhang N. Ding L. Zhang W. Hu J. Zhu S. (2014). Early intra-articular injection of alendronate reduces cartilage changes and subchondral bone loss in rat temporomandibular joints after ovariectomy. Int. J. Oral Maxillofac. Surg. 43 (8), 996–1004. 10.1016/j.ijom.2014.04.003 24811289

[B13] Cheng J. Wang H. Zhang Z. Liang K. (2019). Stilbene glycoside protects osteoblasts against oxidative damage via Nrf2/HO-1 and NF-kappaB signaling pathways. Arch. Med. Sci. 15 (1), 196–203. 10.5114/aoms.2018.79937 30697271 PMC6348355

[B14] Choi M. C. Jo J. Park J. Kang H. K. Park Y. (2019). NF-kappaB signaling pathways in osteoarthritic cartilage destruction. Cells 8. 10.3390/cells8070734 PMC6678954 31319599

[B15] Clevers H. Nusse R. (2012). Wnt/beta-catenin signaling and disease. Cell 149 (6), 1192–1205. 10.1016/j.cell.2012.05.012 22682243

[B16] Clohisy J. C. Hirayama T. Frazier E. Han S. K. Abu-Amer Y. (2004). NF-kB signaling blockade abolishes implant particle-induced osteoclastogenesis. J. Orthop. Res. 22 (1), 13–20. 10.1016/S0736-0266(03)00156-6 14656654

[B17] Danforth K. N. Tworoger S. S. Hecht J. L. Rosner B. A. Colditz G. A. Hankinson S. E. (2007). A prospective study of postmenopausal hormone use and ovarian cancer risk. Br. J. Cancer 96 (1), 151–156. 10.1038/sj.bjc.6603527 17179984 PMC2360221

[B18] Duan P. Bonewald L. F. (2016). The role of the wnt/beta-catenin signaling pathway in formation and maintenance of bone and teeth. Int. J. Biochem. Cell Biol. 77, 23–29. 10.1016/j.biocel.2016.05.015 27210503 PMC4958569

[B19] Ebrahimi S. Alalikhan A. Aghaee-Bakhtiari S. H. Hashemy S. I. (2022). The redox modulatory effects of SP/NK1R system: implications for oxidative stress-associated disorders. Life Sci. 296, 120448. 10.1016/j.lfs.2022.120448 35247438

[B20] Fan R. Pan T. Zhu A. L. Zhang M. H. (2017). Anti-inflammatory and anti-arthritic properties of naringenin via attenuation of NF-kappaB and activation of the heme oxygenase (HO)-1/related factor 2 pathway. Pharmacol. Rep. 69 (5), 1021–1029. 10.1016/j.pharep.2017.03.020 28943290

[B21] Fujita T. Azuma Y. Fukuyama R. Hattori Y. Yoshida C. Koida M. (2004). Runx2 induces osteoblast and chondrocyte differentiation and enhances their migration by coupling with PI3K-Akt signaling. J. Cell Biol. 166 (1), 85–95. 10.1083/jcb.200401138 15226309 PMC2172136

[B22] Gan J. Deng X. Le Y. Lai J. Liao X. (2023). The development of naringin for use against bone and cartilage disorders. Molecules 28. 10.3390/molecules28093716 PMC10180405 37175126

[B23] Gilmore T. D. (2006). Introduction to NF-kappaB: players, pathways, perspectives. Oncogene 25 (51), 6680–6684. 10.1038/sj.onc.1209954 17072321

[B24] Guo Y. Li Y. Xue L. Severino R. P. Gao S. Niu J. (2014). Salvia miltiorrhiza: an ancient Chinese herbal medicine as a source for anti-osteoporotic drugs. J. Ethnopharmacol. 155 (3), 1401–1416. 10.1016/j.jep.2014.07.058 25109459

[B25] Han D. Gao J. Gu X. Hengstler J. G. Zhang L. Shahid M. (2018). P21(Waf1/Cip1) depletion promotes dexamethasone-induced apoptosis in osteoblastic MC3T3-E1 cells by inhibiting the Nrf2/HO-1 pathway. Arch. Toxicol. 92 (2), 679–692. 10.1007/s00204-017-2070-2 28940008

[B26] Han D. Gu X. Gao J. Wang Z. Liu G. Barkema H. W. (2019). Chlorogenic acid promotes the Nrf2/HO-1 anti-oxidative pathway by activating p21(Waf1/Cip1) to resist dexamethasone-induced apoptosis in osteoblastic cells. Free Radic. Biol. Med. 137, 1–12. 10.1016/j.freeradbiomed.2019.04.014 31004750

[B27] Herbst R. S. (2004). Review of epidermal growth factor receptor biology. Int. J. Radiat. Oncol. Biol. Phys. 59 (2), 21–26. 10.1016/j.ijrobp.2003.11.041 15142631

[B28] Huang Q. Meng R. Y. Yang Y. W. Li M. Wang F. Shen W. W. (2017). Protosappanin a inhibits osteoclastogenesis via reducing oxidative stress in RAW264.7 cells. Int. J. Clin. Exp. Pathol. 10 (7), 7498–7510. 31966594 PMC6965276

[B29] Huang W. C. Tsai T. H. Huang C. J. Li Y. Y. Chyuan J. H. Chuang L. T. (2015). Inhibitory effects of wild bitter melon leaf extract on Propionibacterium acnes-induced skin inflammation in mice and cytokine production *in vitro* . Food Funct. 6 (8), 2550–2560. 10.1039/c5fo00550g 26098998

[B30] Ihn H. J. Kim J. A. Lim S. Nam S. H. Hwang S. H. Lim J. (2019). Fermented oyster extract prevents ovariectomy-induced bone loss and suppresses osteoclastogenesis. Nutrients 11. 10.3390/nu11061392 PMC6627411 31234292

[B31] Jaschke N. Hofbauer L. C. Gobel A. Rachner T. D. (2020). Evolving functions of Dickkopf-1 in cancer and immunity. Cancer Lett. 482, 1–7. 10.1016/j.canlet.2020.03.031 32251706

[B32] Kelly R. R. McDonald L. T. Jensen N. R. Sidles S. J. LaRue A. C. (2019). Impacts of psychological stress on osteoporosis: clinical implications and treatment interactions. Front. Psychiatry 10, 200. 10.3389/fpsyt.2019.00200 31024360 PMC6465575

[B33] Kim E. N. Kim G. R. Yu J. S. Kim K. H. Jeong G. S. (2020). Inhibitory effect of (2r)-4-(4-hydroxyphenyl)-2-butanol 2-O-beta-d-apiofuranosyl-(1-->6)-beta-d-glucopyranoside on RANKL-induced osteoclast differentiation and ROS generation in macrophages. Int. J. Mol. Sci. 22. 10.3390/ijms22010222 PMC7795186 33379346

[B34] Kim H. S. Nam S. T. Mun S. H. Lee S. K. Kim H. W. Park Y. H. (2017). DJ-1 controls bone homeostasis through the regulation of osteoclast differentiation. Nat. Commun. 8, 1519. 10.1038/s41467-017-01527-y 29142196 PMC5688089

[B35] Kim S. Y. Cha H. J. Hwangbo H. Park C. Lee H. Song K. S. (2021). Protection against oxidative stress-induced apoptosis by Fermented Sea tangle (laminaria japonica aresch) in osteoblastic mc3t3-E1 cells through activation of Nrf2 signaling pathway. Foods 10. 10.3390/foods10112807 PMC8623046 34829088

[B36] Kong L. Wang B. Yang X. Guo H. Zhang K. Zhu Z. (2017). Picrasidine I from picrasma quassioides suppresses osteoclastogenesis via inhibition of RANKL induced signaling pathways and attenuation of ROS. Prod. Cell Physiol. Biochem. 43 (4), 1425–1435. 10.1159/000481874 29017159

[B37] Lan J. Dou X. Li J. Yang Y. Xue C. Wang C. (2020). l-Arginine ameliorates lipopolysaccharide-induced intestinal inflammation through inhibiting the TLR4/NF-kappaB and MAPK pathways and stimulating beta-defensin expression *in vivo* and *in vitro* . J. Agric. Food Chem. 68 (9), 2648–2663. 10.1021/acs.jafc.9b07611 32064872

[B38] Le Belle J. E. Orozco N. M. Paucar A. A. Saxe J. P. Mottahedeh J. Pyle A. D. (2011). Proliferative neural stem cells have high endogenous ROS levels that regulate self-renewal and neurogenesis in a PI3K/Akt-dependant manner. Cell Stem Cell 8 (1), 59–71. 10.1016/j.stem.2010.11.028 21211782 PMC3018289

[B39] Li Q. Tian C. Q. Liu X. J. Li D. L. Liu H. (2023). Anti-inflammatory and antioxidant traditional Chinese Medicine in treatment and prevention of osteoporosis. Front. Pharmacol. 14. 10.3389/fphar.2023.1203767 PMC10335577 37441527

[B40] Li W. Liu Y. Wang B. Luo Y. Hu N. Chen D. (2016). Protective effect of berberine against oxidative stress-induced apoptosis in rat bone marrow-derived mesenchymal stem cells. Exp. Ther. Med. 12 (6), 4041–4048. 10.3892/etm.2016.3866 28101183 PMC5228215

[B41] Li X. Lin H. Zhang X. Jaspers R. T. Yu Q. Ji Y. (2021). Notoginsenoside R1 attenuates oxidative stress-induced osteoblast dysfunction through JNK signalling pathway. J. Cell Mol. Med. 25 (24), 11278–11289. 10.1111/jcmm.17054 34786818 PMC8650043

[B42] Li Y. P. Wu B. Liang J. Li F. (2019). Isopsoralen ameliorates H2O2-induced damage in osteoblasts via activating the Wnt/beta-catenin pathway. Exp. Ther. Med. 18 (3), 1899–1906. 10.3892/etm.2019.7741 31410152 PMC6676212

[B43] Liu C. Ma R. Wang L. Zhu R. Liu H. Guo Y. (2017). Rehmanniae Radix in osteoporosis: a review of traditional Chinese medicinal uses, phytochemistry, pharmacokinetics and pharmacology. J. Ethnopharmacol. 198, 351–362. 10.1016/j.jep.2017.01.021 28111216

[B44] Liu F. Tan F. Tong W. Fan Q. Ye S. Lu S. (2018). Effect of Zuoguiwan on osteoporosis in ovariectomized rats through RANKL/OPG pathway mediated by beta2AR. Biomed. Pharmacother. 103, 1052–1060. 10.1016/j.biopha.2018.04.102 29710663

[B45] Liu M. Ding H. Wang H. Wang M. Wu X. Gan L. (2021a). Moringa oleifera leaf extracts protect BMSC osteogenic induction following peroxidative damage by activating the PI3K/Akt/Foxo1 pathway. J. Orthop. Surg. Res. 16 (1), 150. 10.1186/s13018-021-02284-x 33610167 PMC7896384

[B46] Liu M. Liu S. Zhang Q. Fang Y. Yu Y. Zhu L. (2021b). Curculigoside attenuates oxidative stress and osteoclastogenesis via modulating Nrf2/NF-kappaB signaling pathway in RAW264.7 cells. J. Ethnopharmacol. 275, 114129. 10.1016/j.jep.2021.114129 33878416

[B47] Liu Y. Hou M. Pan Z. Tian X. Zhao Z. Liu T. (2022). Arctiin-reinforced antioxidant microcarrier antagonizes osteoarthritis progression. J. Nanobiotechnology 20 (1), 303. 10.1186/s12951-022-01505-7 35761235 PMC9235181

[B48] Ma X. Y. Wen X. X. Yang X. J. Zhou D. P. Wu Q. Feng Y. F. (2018). Ophiopogonin D improves osteointegration of titanium alloy implants under diabetic conditions by inhibition of ROS overproduction via Wnt/beta-catenin signaling pathway. Biochimie 152, 31–42. 10.1016/j.biochi.2018.04.022 29705132

[B49] Manolagas S. C. (2010). From estrogen-centric to aging and oxidative stress: a revised perspective of the pathogenesis of osteoporosis. Endocr. Rev. 31 (3), 266–300. 10.1210/er.2009-0024 20051526 PMC3365845

[B50] Martinez-Gil N. Ugartondo N. Grinberg D. Balcells S. (2022). Wnt pathway extracellular components and their essential roles in bone homeostasis. Genes (Basel) 13. 10.3390/genes13010138 PMC8775112 35052478

[B51] McCubrey J. A. Lahair M. M. Franklin R. A. (2006). Reactive oxygen species-induced activation of the MAP kinase signaling pathways. Antioxid. Redox Signal 8 (9-10), 1775–1789. 10.1089/ars.2006.8.1775 16987031

[B52] McMahon M. Campbell K. H. MacLeod A. K. McLaughlin L. A. Henderson C. J. Wolf C. R. (2014). HDAC inhibitors increase NRF2-signaling in tumour cells and blunt the efficacy of Co-adminstered cytotoxic agents. Plos One 9, e114055. 10.1371/journal.pone.0114055 25427220 PMC4245243

[B53] Molagoda I. M. N. Karunarathne W. Choi Y. H. Park E. K. Jeon Y. J. Lee B. J. (2019). Fermented oyster extract promotes osteoblast differentiation by activating the wnt/beta-catenin signaling pathway, leading to bone formation. Biomolecules 9. 10.3390/biom9110711 PMC6920898 31698882

[B54] Ono T. Nakashima T. (2018). Recent advances in osteoclast biology. Histochem Cell Biol. 149 (4), 325–341. 10.1007/s00418-018-1636-2 29392395

[B55] Palikaras K. Lionaki E. Tavernarakis N. (2018). Mechanisms of mitophagy in cellular homeostasis, physiology and pathology. Nat. Cell Biol. 20 (9), 1013–1022. 10.1038/s41556-018-0176-2 30154567

[B56] Portal-Nunez S. Lozano D. Esbrit P. (2012). Role of angiogenesis on bone formation. Histol. Histopathol. 27 (5), 559–566. 10.14670/HH-27.559 22419020

[B57] Qin D. Zhang H. Zhang H. Sun T. Zhao H. Lee W. H. (2019). Anti-osteoporosis effects of osteoking via reducing reactive oxygen species. J. Ethnopharmacol. 244, 112045. 10.1016/j.jep.2019.112045 31260757

[B58] Qin H. Zhao W. Jiao Y. Zheng H. Zhang H. Jin J. (2020). Aqueous extract of Salvia miltiorrhiza bunge-radix puerariae herb pair attenuates osteoporosis in ovariectomized rats through suppressing osteoclast differentiation. Front. Pharmacol. 11, 581049. 10.3389/fphar.2020.581049 33708107 PMC7941748

[B59] Quicke J. G. Conaghan P. G. Corp N. Peat G. (2022). Osteoarthritis year in review 2021: epidemiology & therapy. Osteoarthr. Cartil. 30 (2), 196–206. 10.1016/j.joca.2021.10.003 34695571

[B60] Ramesh P. Jagadeesan R. Sekaran S. Dhanasekaran A. Vimalraj S. (2021). Flavonoids: classification, function, and molecular mechanisms involved in bone remodelling. Front. Endocrinol. (Lausanne) 12, 779638. 10.3389/fendo.2021.779638 34887836 PMC8649804

[B61] Ren Y. L. Song X. M. T. Tan L. Guo C. J. Wang M. Liu H. (2020). A review of the pharmacological properties of psoralen. Front. Pharmacol. 11, 571535. 10.3389/fphar.2020.571535 33013413 PMC7500444

[B62] Rozen N. Bick T. Bajayo A. Shamian B. Schrift-Tzadok M. Gabet Y. (2009). Transplanted blood-derived endothelial progenitor cells (EPC) enhance bridging of sheep tibia critical size defects. Bone 45 (5), 918–924. 10.1016/j.bone.2009.07.085 19665064

[B63] Santos C. X. Raza S. Shah A. M. (2016). Redox signaling in the cardiomyocyte: from physiology to failure. Int. J. Biochem. Cell Biol. 74, 145–151. 10.1016/j.biocel.2016.03.002 26987585

[B64] Scheidereit C. (2006). IkappaB kinase complexes: gateways to NF-kappaB activation and transcription. Oncogene 25 (51), 6685–6705. 10.1038/sj.onc.1209934 17072322

[B65] Schmid T. Brumme U. M. Kemerle S. Zimmer K. (2015). Elderly osteoporosis suspects without diagnosis - interim data from a German geriatric practice. Value Health 18 (7), A654. 10.1016/j.jval.2015.09.2361

[B66] Schroder K. (2019a). NADPH oxidases in bone homeostasis and osteoporosis. Free Radic. Biol. Med. 132, 67–72. 10.1016/j.freeradbiomed.2018.08.036 30189265

[B67] Schroder K. (2019b). Redox control of angiogenesis. Antioxid. Redox Signal 30 (7), 960–971. 10.1089/ars.2017.7429 29665697

[B68] Stohs S. J. Hartman M. J. (2015). Review of the safety and efficacy of Moringa oleifera. Phytother. Res. 29 (6), 796–804. 10.1002/ptr.5325 25808883 PMC6680322

[B69] Strom B. L. Schinnar R. Weber A. L. Bunin G. Berlin J. A. Baumgarten M. (2006). Case-control study of postmenopausal hormone replacement therapy and endometrial cancer. Am. J. Epidemiol. 164 (8), 775–786. 10.1093/aje/kwj316 16997897

[B70] Sun K. Luo J. Guo J. Yao X. Jing X. Guo F. (2020). The PI3K/AKT/mTOR signaling pathway in osteoarthritis: a narrative review. Osteoarthr. Cartil. 28 (4), 400–409. 10.1016/j.joca.2020.02.027 32081707

[B71] Suzuki J. Ogawa M. Muto S. Itai A. Isobe M. Hirata Y. (2011). Novel IkB kinase inhibitors for treatment of nuclear factor-kB-related diseases. Expert Opin. Investig. Drugs 20 (3), 395–405. 10.1517/13543784.2011.559162 21314234

[B72] Talasila K. M. Soentgerath A. Euskirchen P. Rosland G. V. Wang J. Huszthy P. C. (2013). EGFR wild-type amplification and activation promote invasion and development of glioblastoma independent of angiogenesis. Acta Neuropathol. 125 (5), 683–698. 10.1007/s00401-013-1101-1 23429996 PMC3631314

[B73] Tang Y. Jacobi A. Vater C. Zou L. Zou X. Stiehler M. (2015). Icariin promotes angiogenic differentiation and prevents oxidative stress-induced autophagy in endothelial progenitor cells. Stem Cells 33 (6), 1863–1877. 10.1002/stem.2005 25787271

[B74] Tang Y. Vater C. Jacobi A. Liebers C. Zou X. Stiehler M. (2014). Salidroside exerts angiogenic and cytoprotective effects on human bone marrow-derived endothelial progenitor cells via Akt/mTOR/p70S6K and MAPK signalling pathways. Br. J. Pharmacol. 171 (9), 2440–2456. 10.1111/bph.12611 24471788 PMC3997282

[B75] Tao H. Ge G. Liang X. Zhang W. Sun H. Li M. (2020). ROS signaling cascades: dual regulations for osteoclast and osteoblast. Acta Biochim. Biophys. Sin. (Shanghai) 52 (10), 1055–1062. 10.1093/abbs/gmaa098 33085739

[B76] Toledano M. B. (2009). The guardian recruits cops: the p53-p21 axis delegates prosurvival duties to the Keap1-Nrf2 stress pathway. Mol. Cell 34 (6), 637–639. 10.1016/j.molcel.2009.06.005 19560415

[B77] Torrente L. DeNicola G. M. (2022). Targeting NRF2 and its downstream processes: opportunities and challenges. Annu. Rev. Pharmacol. Toxicol. 62, 279–300. 10.1146/annurev-pharmtox-052220-104025 34499527

[B78] Tsumagari K. Abd Elmageed Z. Y. Sholl A. B. Friedlander P. Abdraboh M. Xing M. (2015). Simultaneous suppression of the MAP kinase and NF-kappaB pathways provides a robust therapeutic potential for thyroid cancer. Cancer Lett. 368 (1), 46–53. 10.1016/j.canlet.2015.07.011 26208433 PMC4555189

[B79] Tu Y. Wang K. Liang Y. Jia X. Wang L. Wan J. B. (2019). *Glycine tabacina* ethanol extract ameliorates collagen-induced arthritis in rats via inhibiting pro-inflammatory cytokines and oxidation. J. Ethnopharmacol. 237, 20–27. 10.1016/j.jep.2019.03.035 30880257

[B80] Vurusaner B. Gamba P. Gargiulo S. Testa G. Staurenghi E. Leonarduzzi G. (2016). Nrf2 antioxidant defense is involved in survival signaling elicited by 27-hydroxycholesterol in human promonocytic cells. Free Radic. Biol. Med. 91, 93–104. 10.1016/j.freeradbiomed.2015.12.007 26689473

[B81] Wang Y. K. Huang Z. Q. (2005). Protective effects of icariin on human umbilical vein endothelial cell injury induced by H2O2 *in vitro* . Pharmacol. Res. 52 (2), 174–182. 10.1016/j.phrs.2005.02.023 15967384

[B82] Weaver C. M. Alexander D. D. Boushey C. J. Dawson-Hughes B. Lappe J. M. LeBoff M. S. (2016). Calcium plus vitamin D supplementation and risk of fractures: an updated meta-analysis from the National Osteoporosis Foundation. Osteoporos. Int. 27 (1), 367–376. 10.1007/s00198-015-3386-5 26510847 PMC4715837

[B83] Wei W. Peng C. Gu R. Yan X. Ye J. Xu Z. (2022). Urolithin A attenuates RANKL-induced osteoclastogenesis by co-regulating the p38 MAPK and Nrf2 signaling pathway. Eur. J. Pharmacol. 921, 174865. 10.1016/j.ejphar.2022.174865 35231470

[B84] Wong P. Lv Z. Li J. Wei Q. Xu L. Fang B. (2022). A novel RANKL-targeted furoquinoline alkaloid ameliorates bone loss in ovariectomized osteoporosis through inhibiting the NF-kappaB signal pathway and reducing reactive oxygen species. Oxid. Med. Cell Longev. 2022, 5982014. 10.1155/2022/5982014 36388169 PMC9652067

[B85] Wu Y. Gao L. J. Fan Y. S. Chen Y. Li Q. (2021). Network pharmacology-based analysis on the action mechanism of oleanolic acid to alleviate osteoporosis. ACS Omega 6 (42), 28410–28420. 10.1021/acsomega.1c04825 34723038 PMC8552458

[B86] Xi H. R. Ma H. P. Yang F. F. Gao Y. H. Zhou J. Wang Y. Y. (2018). Total flavonoid extract of Epimedium herb increases the peak bone mass of young rats involving enhanced activation of the AC10/cAMP/PKA/CREB pathway. J. Ethnopharmacol. 223, 76–87. 10.1016/j.jep.2018.05.023 29783019

[B87] Xiao L. Zhong M. Huang Y. Zhu J. Tang W. Li D. (2020). Puerarin alleviates osteoporosis in the ovariectomy-induced mice by suppressing osteoclastogenesis via inhibition of TRAF6/ROS-dependent MAPK/NF-kappaB signaling pathways. Aging (Albany NY) 12 (21), 21706–21729. 10.18632/aging.103976 33176281 PMC7695364

[B88] Xu Q. Chen G. Liu X. Dai M. Zhang B. (2019). Icariin inhibits RANKL-induced osteoclastogenesis via modulation of the NF-kappaB and MAPK signaling pathways. Biochem. Biophys. Res. Commun. 508 (3), 902–906. 10.1016/j.bbrc.2018.11.201 30538045

[B89] Yabas M. Orhan C. Er B. Tuzcu M. Durmus A. S. Ozercan I. H. (2021). A next generation formulation of curcumin ameliorates experimentally induced osteoarthritis in rats via regulation of inflammatory mediators. Front. Immunol. 12, 609629. 10.3389/fimmu.2021.609629 33776996 PMC7994281

[B90] Yang Y. H. Chen K. Li B. Chen J. W. Zheng X. F. Wang Y. R. (2013). Estradiol inhibits osteoblast apoptosis via promotion of autophagy through the ER-ERK-mTOR pathway. Apoptosis 18 (11), 1363–1375. 10.1007/s10495-013-0867-x 23743762

[B91] Yao Q. Wu X. H. Tao C. Gong W. Y. Chen M. J. Qu M. H. (2023). Osteoarthritis: pathogenic signaling pathways and therapeutic targets. Signal Transduct. Target. Ther. 8, 56. 10.1038/s41392-023-01330-w 36737426 PMC9898571

[B92] Zhai Z. J. Li H. W. Liu G. W. Qu X. H. Tian B. Yan W. (2014). Andrographolide suppresses RANKL-induced osteoclastogenesis *in vitro* and prevents inflammatory bone loss *in vivo* . Br. J. Pharmacol. 171 (3), 663–675. 10.1111/bph.12463 24125472 PMC3969079

[B93] Zhang J. K. Yang L. Meng G. L. Fan J. Chen J. Z. He Q. Z. (2012). Protective effect of tetrahydroxystilbene glucoside against hydrogen peroxide-induced dysfunction and oxidative stress in osteoblastic MC3T3-E1 cells. Eur. J. Pharmacol. 689 (1-3), 31–37. 10.1016/j.ejphar.2012.05.045 22683865

[B94] Zhang Y. H. Shangguan W. J. Zhao Z. J. Zhou F. C. Liu H. T. Liang Z. H. (2022). Naringin inhibits apoptosis induced by cyclic stretch in rat annular cells and partially attenuates disc degeneration by inhibiting the ROS/NF-kappaB pathway. Oxid. Med. Cell Longev. 2022, 6179444. 10.1155/2022/6179444 35251479 PMC8890877

[B95] Zhao B. Peng Q. Wang D. Zhou R. Wang R. Zhu Y. (2022). Leonurine protects bone mesenchymal stem cells from oxidative stress by activating mitophagy through PI3K/Akt/mTOR pathway. Cells 11. 10.3390/cells11111724 PMC9179429 35681421

[B96] Zhao Y. Zheng Z. Zhang M. Wang Y. Hu R. Lin W. (2021). Design, synthesis, and evaluation of mono-carbonyl analogues of curcumin (MCACs) as potential antioxidants against periodontitis. J. Periodontal Res. 56 (4), 656–666. 10.1111/jre.12862 33604902

[B97] Zhou T. Gai Z. Gao X. Li L. (2021). The potential mechanism of exercise combined with natural extracts to prevent and treat postmenopausal osteoporosis. J. Healthc. Eng. 2021, 2852661. 10.1155/2021/2852661 34956564 PMC8709765

[B98] Zhu Y. Z. Wu W. Zhu Q. Liu X. (2018). Discovery of Leonuri and therapeutical applications: from bench to bedside. Pharmacol. Ther. 188, 26–35. 10.1016/j.pharmthera.2018.01.006 29360539

[B99] Zou Z. Chang H. Li H. Wang S. (2017). Induction of reactive oxygen species: an emerging approach for cancer therapy. Apoptosis 22 (11), 1321–1335. 10.1007/s10495-017-1424-9 28936716

